# Supplementary material for: Proteomic Profiling of Paracoccidioides brasiliensis in Response to Phenacylideneoxindol Derivative: Unveiling Molecular Targets and Pathways
Source: J Fungi (Basel). 2023 Aug 16;9(8):854. doi: 10.3390/jof9080854 (PMC10455990; doi:10.3390/jof9080854)

Proteomic Profiling of *Paracoccidioides brasiliensis* in Response to Phenacylideneoxindol Derivative: Unveiling Molecular Targets and Pathways

Supplementary Material

Table S1. *P. brasiliensis* proteins downregulated after incubation with OPI

**Accession *P. brasiliensis* (*Pb*18) (a)**	**Protein**	**EC Number (b)**	**Score**	**Fold change**	**TRAT:CTRL_P**
**Metabolism**					
Amino acid metabolism					
PADG_12043	cysteine desulfurase mitochondrial	2.8.1.7	648.21	CTRL	
PADG_01963	glycine cleavage system H protein	2.1.2.10	1121.2	CTRL	
PADG_01536	glutamine synthetase	6.3.1.2	361.31	CTRL	
PADG_02456	cystathionine gamma-lyase	4.4.1.1	455.01	CTRL	
PADG_05492	serine 3-dehydrogenase	1.1.1.276	4170.9	0.7	0
PADG_03886	lysine--tRNA ligase cytoplasmic	6.1.1.6	534.33	CTRL	
PADG_06144	saccharopine dehydrogenase	1.5.1.10	473.85	CTRL	
PADG_08468	4-hydroxyphenylpyruvate dioxygenase	1.13.11.27	2086.1	0.7	0
PADG_07366	methylcrotonoyl-CoA carboxylase subunit alpha	6.4.1.4	1164.5	0.7	0
Lipid metabolism					
PADG_05783	farnesyl pyrophosphate synthetase	2.5.1.10	2083.9	CTRL	
PADG_03199	ergosterol biosynthesis protein Erg 28		338.8	CTRL	
PADG_00255	fatty acid synthase subunit beta	2.3.1.86	656.91	CTRL	
PADG_00254	fatty acid synthase subunit alpha	2.3.1.86	557.26	CTRL	
PADG_00546	3-oxoacyl-acyl-carrier protein reductase	1.1.1.100	1008.2	CTRL	
PADG_02244	acyl-CoA dehydrogenase	1.3.8.1	522.55	CTRL	
C-compound and carbohydrate metabolism					
PADG_04103	pyruvate carboxylase	6.4.1.1	382.66	CTRL	
PADG_03825	NAD-specific glutamate dehydrogenase	1.4.1.2	411.54	CTRL	
PADG_05217	acetyltransferase	2.3.1.5.	577.98	CTRL	
Nucleotide metabolism					
PADG_02246	adenosine kinase	2.7.1.20	574.24	CTRL	
**Energy**					
Electron transport and membrane-associated energy conservation					
PADG_04501	ubiquinol-cytochrome c reductase subunit 7	1.10.2.2	652.51	CTRL	

PADG_11981	V-type proton ATPase catalytic subunit A	3.6.3.14	442.31	CTRL	
PADG_08394	cytochrome b-c1 complex subunit 2	1.10.2.2	1030.1	CTRL	
PADG_03163	cytochrome c peroxidase. mitochondrial	1.11.1.5	4873.6	0.6	0
PADG_07964	vacuolar ATP synthase subunit E	3.6.3.14	724.14	CTRL	
Tricarboxylic acid pathway					
PADG_02260	succinyl-CoA ligase subunit alpha	6.2.1.5	3349.1	0.4	0
PADG_04993	citrate synthase subunit 1	2.3.3.8	776.32	CTRL	
PADG_07475	succinate dehydrogenase	1.3.5.11	416.4	CTRL	
Glycolysis and gluconeogenesis					
PADG_01896	phosphoglycerate kinase	2.7.2.3	2512.9	0.7	0
PADG_03631	phosphoglycerate mutase	5.4.2.12	491.45	CTRL	
**Protein synthesis**					
PADG_07891	ubiquitin-60S ribosomal protein L40		2351.7	0.3	0
PADG_01605	polyubiquitin		2628.7	0.3	0
PADG_00995	ubiquitin-40S ribosomal protein S27a		2542.1	0.5	0
PADG_08033	eukaryotic translation initiation factor 3 subunit B		506.4	CTRL	
PADG_00692	elongation factor 1-alpha		7312.7	0.6	0
PADG_08125	elongation factor 2		6751.1	0.7	0
PADG_01949	translation elongation factor Tu		9146.6	0.8	0
**Biogenesis of cellular components**					
PADG_03895	dynein light chain. cytoplasmic		5013.1	0.6	0
PADG_00912	UDP-galactopyranose mutase	5.4.99.9	618.57	CTRL	
PADG_03959	ARP2/3 actin-organizing complex subunit Sop2		428.53	CTRL	
**Cellular transport, transport facilitation and transport routes**					
PADG_07508	phosphatidylinositol transfer protein SFH5		1573.6	0.6	0
**Cell rescue, defense and virulence**					
PADG_00324	catalase	1.11.1.6	5493.8	0.7	0
**Cell cycle and DNA processing**					
PADG_04810	GTP-binding nuclear protein GSP1/Ran		3937.9	0.7	0
PADG_05683	cell division control protein 48		4405.6	0.7	0
PADG_08483	heterochromatin protein HP1		481.98	CTRL	
**Transcription**					
PADG_04311	cellular nucleic acid-binding protein		1550	0.7	0
PADG_02752	116 kDa U5 small nuclear ribonucleoprotein component		418.17	CTRL	

**Protein fate (folding, modification, destination)**					
PADG_04092	peptidyl-prolyl cis-trans isomerase B	5.2.1.8	1147.9	CTRL	
**Unclassified**					
PADG_04312	hypothetical protein		529.74	0.7	0
PADG_07274	hypothetical protein		958.34	0.8	0
PADG_08368	hypothetical protein		719.16	CTRL	
PADG_08480	hypothetical protein		992	CTRL	
PADG_03660	hypothetical protein		790.74	CTRL	

a. Functional classification based on the functional category database Munich Information Center for Protein Sequences (MIPS) and access numbers of the *P. brasiliensis* proteins available in the database (http://www.broadinstitute.org/annotation/genome/*Paracoccidioides_brasiliensis*/MultiHome.html).

b. Ec number. Enzyme access number available in the Brenda-enzyme database.

CTRL- identified only in the control condition.

TRAT:CTRL_P: statistical analysis with ExpressionE informatics v.2.5.2

Table S2. ***P. brasiliensis*** **proteins upregulated after incubation with OPI**

**Accession *P. brasiliensis* (*Pb*18) (a)**	**Protein**	**EC Number (b)**	**Score**	**Fold change**	**TRAT:CTRL_P**
**Metabolism**					
Amino acid metabolism					
PADG_02914	glycine cleavage system T protein	2.1.2.10	1121.61	1.3	1
PADG_00210	glycine dehydrogenase	1.4.1.10	1966.59	1.4	1
PADG_06671	3-isopropylmalate dehydrogenase A	1.1.1.85	539.43	1.3	1
PADG_05897	serine-tRNA ligase	6.1.1.11	532.95	TRAT	
PADG_05277	serine hydroxymethyltransferase	2.1.2.1	4289.63	1.4	1
PADG_08406	*o*-acetylhomoserine (thiol)-lyase	2.5.1.49	5334.69	1.4	1
PADG_00663	homoserine dehydrogenase	1.1.1.3	605.33	1.6	1
PADG_02777	threonine synthase	4.2.3.1	594.78	TRAT	
PADG_03859	L-threonine 3-dehydrogenase	1.1.1.103	480.41	TRAT	
PADG_01404	aspartate aminotransferase	2.6.1.1	1407.03	1.3	1
PADG_08376	aspartate-semialdehyde dehydrogenase	1.2.1.11	2962.97	1.4	1
PADG_05085	1-pyrroline-5-carboxylate dehydrogenase	1.2.1.88	3033.52	1.3	1
PADG_08466	homogentisate 1,2-dioxygenase	1.13.11.5	797.35	1.3	1
PADG_02214	4-aminobutyrate aminotransferase	2.6.1.96	6943.21	1.4	1
PADG_03466	3-hydroxyisobutyrate dehydrogenase	1.1.1.31	7470.5	1.4	1
PADG_01928	S-adenosylmethionine synthase	2.5.1.6	2205.46	1.4	1
PADG_04570	branched-chain amino acid aminotransferase	2.6.1.42	3990.02	1.4	1
PADG_01328	ornithine aminotransferase	2.6.1.13	1255.02	1.5	1
PADG_00402	betaine aldehyde dehydrogenase	1.2.1.8	634.37	1.4	1
PADG_01286	homoisocitrate dehydrogenase	1.1.1.286	850.26	1.4	1
C-compound and carbohydrate metabolism					
PADG_06494	dihydrolipoyl dehydrogenase	1.8.1.4	3809.74	1.3	1
PADG_02145	glycogen phosphorylase	2.4.1.1	909.62	1.5	1
PADG_07674	carbonic anhydrase	4.2.1.1	1665.23	1.4	1

Lipid metabolism					
PADG_06382	acetyl-CoA acetyltransferase	2.3.1.9	1846.05	1.3	1
Nitrogen and sulfur metabolism					
PADG_06490	formamidase	3.5.1.49	4314.92	1.5	1
Nucleotide metabolism					
PADG_07585	inosine-5'-monophosphate dehydrogenase	1.1.1.205	618.56	1.3	1
PADG_01100	uracil phosphoribosyltransferase	2.4.2.9	1768.93	1.4	1
PADG_00988	ribonuclease T2	3.1.27.1	514.48	TRAT	
PADG_04828	adenylosuccinate lyase	4.3.2.2	904.14	1.5	1
Phosfate metabolism					
PADG_04175	inorganic pyrophosphatase	3.6.1.1	2895.66	1.3	1
**Energy**					
Glycolysis and gluconeogenesis					
PADG_03813	hexokinase	2.7.1.1	3726.23	1.3	1
PADG_01278	pyruvate kinase	2.7.1.40	11269.6	1.3	1
PADG_08503	phosphoenolpyruvate carboxykinase	4.1.1.49	960.1	1.4	1
Tricarboxylic acid pathway					
PADG_08119	fumarate hydratase	4.2.1.2	1604.83	1.3	1
PADG_07210	malate dehydrogenase	1.1.1.37	28764	1.3	1
PADG_05904	dihydrolipoamide succinyltransferase	2.3.1.61	1101.68	1.3	1
Glyoxylate cycle					
PADG_01483	isocitrate lyase	4.1.3.1	1955.1	1.3	1
Methyl citrate cycle					
PADG_04710	2-methylcitrate synthase	2.3.3.5	8409.58	1.3	1
Fermentation					
PADG_11405	alcohol dehydrogenase 1	1.1.1.1	969.2	1.4	1
Respiration					
PADG_06515	suaprga1		24981.7	1.3	1
Electron transport and membrane-associated energy conservation					
PADG_08349	ATP synthase subunit beta mitochondrial	3.6.3.14	25661.9	1.3	1
PADG_07789	ATP synthase subunit delta mitochondrial	3.6.3.14	1832.37	TRAT	
PADG_04729	ATP synthase subunit D mitochondrial	3.6.3.14	2432.08	1.5	1
PADG_05750	cytochrome c oxidase polypeptide VI	1.9.3.1	4605.58	1.5	1
PADG_06978	cytochrome c		2402.86	1.4	1
PADG_07749	NAD(P)H:quinone oxidoreductase. type IV	1.6.5.2	61735	1.4	1
PADG_01366	NADH-ubiquinone oxidoreductase	1.6.5.2	871.23	1.4	1

PADG_00688	F-type H+-transporting ATPase subunit H	3.6.3.14	2753.58	1.6	1
**Cell fate**					
PADG_08615	tropomyosin-1		7420.29	1.3	1
**Protein synthesis**					
PADG_08715	ribosomal protein L28e		8600.58	1.4	1
PADG_02056	ribosomal protein L7/L12		8745.29	1.3	1
PADG_01654	40S ribosomal protein S6-A		9211.99	1.3	1
PADG_00784	40S ribosomal protein S0		17982.4	1.4	1
PADG_03326	40S ribosomal protein S9		5550.94	1.3	1
PADG_03315	40S ribosomal protein S4		3012.44	1.4	1
PADG_00333	40S ribosomal protein S16		5431.62	1.4	1
PADG_00354	40S ribosomal protein S17		6613.62	1.4	1
PADG_02445	40S ribosomal protein S15		30663.5	1.4	1
PADG_08602	40S ribosomal protein S2		10459.7	1.4	1
PADG_07685	40S ribosomal protein		4007.36	1.4	1
PADG_06838	40S ribosomal protein S5		7030.37	1.4	1
PADG_00942	40S ribosomal protein S7		17451.1	1.4	1
PADG_06680	40S ribosomal protein S22		6891.2	1.6	1
PADG_12365	40S ribosomal protein S8-A		2025.18	1.4	1
PADG_01407	40S ribosomal protein		7203.81	1.3	1
PADG_11379	60S ribosomal protein L5		5854.33	1.3	1
PADG_00514	60S ribosomal protein L16		2428.02	1.3	1
PADG_02828	60S ribosomal protein L10a		2307.15	1.3	1
PADG_04588	60S ribosomal protein L22		8081.96	1.3	1
PADG_05939	60S ribosomal protein L27a		1335.61	1.4	1
PADG_00612	60S ribosomal protein L27-A		3385.56	1.4	1
PADG_06726	60S ribosomal protein L17		2076.97	1.5	1
PADG_03781	60S ribosomal protein L30		3182.43	1.4	1
PADG_03325	60S ribosomal protein L21-A		11439	1.4	1
PADG_04848	60S ribosomal protein L8-B		1887.85	1.4	1
PADG_04449	60S ribosomal protein L23		3782.12	1.5	1
PADG_05883	60S ribosomal protein L25		15591.8	1.5	1
PADG_07924	60S ribosomal protein L24		681.22	1.5	1
PADG_03873	60S ribosomal protein L20		3445.41	1.5	1
PADG_03856	60S ribosomal protein L15		3750.28	1.4	1
PADG_03778	60S ribosomal protein L10-A		5593.24	1.5	1
PADG_04106	60S ribosomal protein L11		1323.36	1.5	1
PADG_02249	60S ribosomal protein L2		3203.24	1.5	1
PADG_05338	60S ribosomal protein L18-B		4233.38	1.5	1
PADG_01083	60S ribosomal protein L32		1552.35	1.5	1
PADG_01387	60S ribosomal protein L7		1916.87	1.6	1
PADG_11832	60S ribosomal protein L31		2077.8	1.6	1
PADG_05721	60S ribosomal protein L4-A		5906.33	1.5	1

PADG_04402	60S ribosomal protein L34-A		2147.49	TRAT	
PADG_08244	60S acidic ribosomal protein P1		19823.1	1.3	1
PADG_06110	translation initiation factor SUI1		5317.54	1.5	1
PADG_04057	translation initiation factor 3 subunit		2275.88	1.4	1
PADG_11904	translational activator		551.15	1.5	1
PADG_07803	protein transporter SEC23		6462.38	1.3	1
PADG_01530	guanine nucleotide-binding protein subunit beta-like protein		4123.37	1.3	1
**Protein fate (folding, modification, destination)**					
PADG_03221	thimet oligopeptidase		1082.29	1.4	1
PADG_06992	mitochondrial co-chaperone Grp		4746.56	1.6	1
PADG_12323	peptidyl-prolyl cis-trans isomerase	5.2.1.8	1332.36	1.4	1
**Cellular communication/signal transduction**					
PADG_02017	calmodulin		13307	1.3	1
PADG_05517	rho GDP-dissociation inhibitor		997.58	TRAT	
**Cell cycle and DNA processing**					
PADG_01032	DNA-binding protein 42 kDa		544.02	1.3	1
PADG_05906	histone H2A		485.87	TRAT	
**Transcription**					
PADG_02555	nucleic acid-binding protein		2568.2	1.4	1
PADG_03696	nuclear polyadenylated RNA-binding protein Nab2		772.17	1.4	1
**Biogenesis of cellular components**					
PADG_04761	mannosyl-oligosaccharide glucosidase	3.2.1.106	618.03	1.5	1
**Cell Rescue, Defense and virulence**					
PADG_01711	Hsp90 co-chaperone AHA1		701.31	TRAT	
PADG_01954	superoxide dismutase	1.15.1.1	2101.82	1.4	1
**Unclassified**					
PADG_08270	hypothetical protein		904.53	1.3	1
PADG_07064	hypothetical protein		928.74	1.3	1
PADG_02338	hypothetical protein		780.78	4.1	1
PADG_03654	hypothetical protein		1385.83	TRAT	
PADG_01900	hypothetical protein		812.3	TRAT	
PADG_08152	hypothetical protein		2935.69	TRAT	
PADG_01773	hypothetical protein		685.72	TRAT	
PADG_04389	hypothetical protein		687.61	TRAT	
PADG_00541	hypothetical protein		1892.82	TRAT	
PADG_03210	hypothetical protein		2911.28	1.7	1

PADG_05034	hypothetical protein	1240.88	1.4	1
PADG_05703	hypothetical protein	3286.77	1.4	1
PADG_02764	hypothetical protein	1358.94	1.5	1
PADG_11227	hypothetical protein	992.21	1.4	1
PADG_11950	hypothetical protein	2730.66	1.3	1
PADG_02343	hypothetical protein	1200.84	1.4	1
PADG_02981	hypothetical protein	8121.04	2.3	1
PADG_08724	hypothetical protein	1946.17	TRAT	
PADG_11865	hypothetical protein	388.26	1.5	1

a. Functional classification based on the functional category database Munich Information Center for Protein Sequences (MIPS) and access numbers of the *P. brasiliensis* proteins available in the database (http://www.broadinstitute.org/annotation/genome/*Paracoccidioides_brasiliensis*/MultiHome.html).
b. Ec number. Enzyme access number available in the Brenda-enzyme database.
TRAT- identified only in the treated condition.
TRAT:CTRL_P: statistical analysis with ExpressionE informatics v.2.5.2

Table S3. Proteins that were not differentially expressed considering a fold change of 1.3

Accession Number	Protein	Score	Fold change
PADG_07884	polyadenylate-binding protein. cytoplasmic and nuclear	885.63	0.77
PADG_04439	hypothetical protein	4590.35	0.77
PADG_00694	hypothetical protein	567.51	0.77
PADG_06568	hypothetical protein	16108.55	0.79
PADG_04374	hypothetical protein	1832.5	0.79
PADG_05837	E3 ubiquitin ligase complex SCF subunit sconC	4456.05	0.81
PADG_12077	actin	789.67	0.81
PADG_03983	6.7-dimethyl-8-ribityllumazine synthase	603.74	0.82
PADG_07715	hsp90-like protein	3039.57	0.82
PADG_06740	hypothetical protein	11486.47	0.83
PADG_05032	hypothetical protein	6046.13	0.85
PADG_06265	hypothetical protein	7192.99	0.86
PADG_04056	14-3-3 family protein epsilon	4295.13	0.86
PADG_07213	pyruvate dehydrogenase complex dihydrolipoamide acetyltransferase	6996.61	0.87
PADG_03602	hypothetical protein	755.69	0.89
PADG_03118	hypothetical protein	4489.61	0.89
PADG_00001	peptidyl-prolyl cis-trans isomerase H	1895.71	0.90
PADG_07460	hypothetical protein	450.34	0.90
PADG_02048	hypothetical protein	959.86	0.91
PADG_00872	histone	2696.73	0.91
PADG_02683	UV excision repair protein Rad23	873.89	0.92
PADG_06876	hypothetical protein	2816.15	0.93
PADG_03651	6-phosphogluconate dehydrogenase. decarboxylating 1	1147.18	0.94
PADG_00446	hypothetical protein	2124	0.94
PADG_04709	methyl-isocitrate lyase	481.54	0.95
PADG_04559	hypothetical protein	2655.86	0.95
PADG_04440	hypothetical protein	19787.8	0.95
PADG_05504	thioredoxin	7773.85	0.95
PADG_00067	hypothetical protein	542.84	0.95

PADG_03176	hypothetical protein	1154.4	0.96
PADG_02063	pyruvate dehydrogenase (acetyl-transferring) E1 component. alpha subunit	1144.22	0.97
PADG_05907	histone H2B	2186.32	0.97
PADG_03963	hypothetical protein	12996.71	0.98
PADG_11468	hypothetical protein	879.4	0.99
PADG_06033	hypothetical protein	1200.62	0.99
PADG_00714	pyruvate decarboxylase	5441.92	1
PADG_02896	hypothetical protein	14319.67	1.01
PADG_07023	hypothetical protein	1307.38	1.02
PADG_00451	glucose-6-phosphate isomerase	2802.51	1.02
PADG_03073	nuclear movement protein nudC	3925.42	1.02
PADG_06313	40S ribosomal protein S18	6023.22	1.02
PADG_00246	hypothetical protein	5879.92	1.02
PADG_03403	hypothetical protein	929.09	1.03
PADG_07627	hypothetical protein	1990.57	1.04
PADG_02592	hypothetical protein	5277.75	1.04
PADG_01427	40S ribosomal protein S12	2464.37	1.05
PADG_02751	hypothetical protein	3567.94	1.05
PADG_04657	nascent polypeptide-associated complex subunit beta	4461.65	1.06
PADG_07420	transaldolase	7526.31	1.06
PADG_06165	hypothetical protein	337.09	1.06
PADG_03967	hypothetical protein	4626.93	1.07
PADG_07946	hypothetical protein	1528.69	1.07
PADG_07014	hypothetical protein	666.02	1.08
PADG_06429	ketol-acid reductoisomerase. mitochondrial	2013.72	1.09
PADG_00171	hypothetical protein	1650.2	1.09
PADG_07435	hypothetical protein	2020.88	1.09
PADG_05822	pyridoxine biosynthesis protein pyroA	3431.83	1.10
PADG_08118	hsp72-like protein	45613.76	1.10
PADG_00615	hypothetical protein	2109.09	1.10
PADG_06048	40S ribosomal protein S27	5422.03	1.10
PADG_01363	hypothetical protein	3205.7	1.10
PADG_03522	methylthioadenosine phosphorylase	702.62	1.10
PADG_01486	hypothetical protein	1916.53	1.11
PADG_01914	60S ribosomal protein L35	1816.06	1.11
PADG_07081	hypothetical protein	4857.25	1.11
PADG_00443	dihydropteroate synthase	2502.89	1.11

PADG_08369	hsp60-like protein	41789.88	1.11
PADG_05109	2.3-bisphosphoglycerate-independent phosphoglycerate mutase	1614.42	1.12
PADG_01564	methylmalonate-semialdehyde dehydrogenase (acylating)	3545.25	1.12
PADG_04687	hypothetical protein	1114.11	1.12
PADG_02446	60S acidic ribosomal protein P2	8778.9	1.12
PADG_01621	hypothetical protein	7546.76	1.12
PADG_11845	aconitate hydratase. mitochondrial	4217.13	1.12
PADG_02030	hypothetical protein	4687.51	1.13
PADG_02761	hsp75-like protein	4000.97	1.13
PADG_06488	hypothetical protein	13316.72	1.13
PADG_03194	hypothetical protein	1284.01	1.15
PADG_04702	malate synthase. glyoxysomal	1784.29	1.15
PADG_04076	hypothetical protein	2362.57	1.15
PADG_01228	hypothetical protein	1860.56	1.15
PADG_01551	thioredoxin reductase	704.65	1.15
PADG_02967	hypothetical protein	1082.05	1.15
PADG_02733	hypothetical protein	865.48	1.15
PADG_01718	saccharopine dehydrogenase [NADP+. L-glutamate-forming] (451 aa)	725.39	1.16
PADG_04067	proteasome component PUP3	1576.81	1.16
PADG_03841	protein disulfide-isomerase domain	8600.23	1.16
PADG_06182	hypothetical protein	398.86	1.16
PADG_04288	hypothetical protein	37903.7	1.17
PADG_07370	hypothetical protein	1387.63	1.17
PADG_04379	hypothetical protein	8220.83	1.17
PADG_01886	adenosylhomocysteinase	2562.42	1.17
PADG_04167	hypothetical protein	641.23	1.17
PADG_07249	hypothetical protein	1121.98	1.17
PADG_02833	ADP-ribosylation factor	1627.49	1.18
PADG_05111	serine hydroxymethyltransferase. cytosolic	3032.21	1.18
PADG_01558	hypothetical protein	931.55	1.18
PADG_11679	proliferating cell nuclear antigen (pcna)	2504.93	1.19
PADG_00331	uricase	558.91	1.19
PADG_08465	fumarylacetoacetase	2722.05	1.19
PADG_05922	hypothetical protein	3468.32	1.19
PADG_02637	hypothetical protein	1818.95	1.19
PADG_00430	hsp7-like protein	15369.86	1.19

PADG_01372	mannitol-1-phosphate 5-dehydrogenase	5035.63	1.19
PADG_04718	2-methylcitrate dehydratase	18552.45	1.20
PADG_08604	ATP phosphoribosyltransferase	398.66	1.20
PADG_02625	hypothetical protein	861.38	1.20
PADG_04603	spermidine synthase	753.68	1.20
PADG_02785	hypothetical protein	15005.18	1.20
PADG_02759	hypothetical protein	6292.38	1.20
PADG_02728	hypothetical protein	1180.75	1.20
PADG_05523	hypothetical protein	1418.88	1.20
PADG_05025	ribosomal protein L24	2528.57	1.22
PADG_12426	1.4-alpha-glucan-branching enzyme	1394.58	1.22
PADG_02888	hypothetical protein	6092.82	1.22
PADG_08464	maleylacetoacetate isomerase	1597.59	1.22
PADG_00183	hypothetical protein	2250.59	1.22
PADG_02652	hypothetical protein	18057.45	1.22
PADG_02183	hypothetical protein	1038.17	1.22
PADG_07953	peptidyl-prolyl cis-trans isomerase-like 1	10047.4	1.22
PADG_06502	40S ribosomal protein S20	2053.34	1.22
PADG_11413	hypothetical protein	4183.77	1.22
PADG_02484	valine-tRNA ligase	609.7	1.22
PADG_04636	hypothetical protein	4936.58	1.22
PADG_07369	hypothetical protein	5310.21	1.23
PADG_01867	hypothetical protein	1253.83	1.23
PADG_02411	glyceraldehyde-3-phosphate dehydrogenase	36468.04	1.23
PADG_05587	hypothetical protein	649.68	1.23
PADG_04939	hypothetical protein	1879.78	1.23
PADG_01687	hypothetical protein	11191.74	1.23
PADG_03020	hypothetical protein	731.32	1.23
PADG_02735	proteasome component PRE6	1212.33	1.23
PADG_04730	nascent polypeptide-associated complex subunit alpha	1976.8	1.24
PADG_07813	ATP synthase F1. gamma subunit	8105.43	1.24
PADG_04099	phosphoribosylaminoimidazolecarboxamide formyltransferase/IMP cyclohydrolase	4942.01	1.24
PADG_11132	phosphoglucomutase	1049.48	1.24
PADG_01209	hypothetical protein	10077.34	1.24
PADG_07606	hypothetical protein	3994.21	1.24
PADG_00734	urea carboxylase	433.01	1.24

PADG_08387	citrate synthase. mitochondrial	4962.74	1.24
PADG_06997	hypothetical protein	2058.65	1.24
PADG_06906	triosephosphate isomerase	8002.14	1.25
PADG_00824	hypothetical protein	1699.66	1.25
PADG_07524	nucleoside diphosphate kinase	32664.7	1.25
PADG_06766	mitochondrial-processing peptidase subunit beta	1423.17	1.25
PADG_00335	40S ribosomal protein S14	6224.66	1.25
PADG_00668	fructose-bisphosphate aldolase 1	15783.66	1.25
PADG_06599	40S ribosomal protein S25	2152.11	1.25
PADG_06525	40S ribosomal protein S1	5119.54	1.25
PADG_00422	hypothetical protein	643.53	1.25
PADG_08054	malate dehydrogenase. NAD-dependent	8786.75	1.25
PADG_05081	aldehyde dehydrogenase	10549.69	1.25
PADG_00060	hypothetical protein	755.97	1.25
PADG_00849	hypothetical protein	786.21	1.27
PADG_12253	60S ribosomal protein L3	4213.76	1.27
PADG_01267	40S ribosomal protein S11	3346.82	1.27
PADG_03276	S-(hydroxymethyl)glutathione dehydrogenase	1096.19	1.27
PADG_04315	hypothetical protein	2642.68	1.27
PADG_04165	hypothetical protein	1004.75	1.27
PADG_03686	hypothetical protein	563.95	1.27
PADG_02561	ATP synthase subunit alpha. mitochondrial	18216.72	1.28
PADG_08605	40S ribosomal protein S28	16022.18	1.28
PADG_01762	oxoglutarate dehydrogenase (succinyl-transferring). E1 component	685.2	1.28
PADG_01755	hypothetical protein	1413.37	1.28
PADG_00656	non-histone chromosomal protein 6	8808.26	1.28
PADG_05160	hypothetical protein	567.81	1.28
PADG_04059	enolase	44398.99	1.28
PADG_06546	hypothetical protein	808.86	1.28
PADG_05798	hypothetical protein	8506.6	1.28
PADG_12025	hypothetical protein	413.78	1.28
PADG_03203	hypothetical protein	789.03	1.28
PADG_03562	chaperone DnaK	6243.46	1.28
PADG_06805	hypothetical protein	5433.17	1.28
PADG_06319	glutamate decarboxylase	621.88	1.28

PADG_08013	succinate dehydrogenase [ubiquinone] iron-sulfur subunit. mitochondrial	1079.23	1.28
PADG_04249	isocitrate dehydrogenase [NADP]. mitochondrial	2835.57	1.29
PADG_02825	hypothetical protein	532.65	1.29
PADG_01706	hypothetical protein	2061.44	1.29
PADG_05947	nicotinate-nucleotide diphosphorylase (carboxylating)	2977.39	1.29
PADG_08238	40S ribosomal protein S26E	1470.93	1.29
PADG_07422	hypothetical protein	751.24	1.29
PADG_07782	deoxyuridine 5'-triphosphate nucleotidohydrolase	10921.27	1.29
PADG_04604	transketolase	1840.39	1.29
PADG_08328	5-methyltetrahydropteroyltriglutamate-homocysteine S-methyltransferase	4767.2	1.29
PADG_00052	succinate dehydrogenase [ubiquinone] flavoprotein subunit. mitochondrial	1905.65	1.29

Table S4. Protein related with Defense and virulence ROS, Membrane cell and Fermentation process

**Accession *P. brasiliensis* (*Pb*18) (a)**	**Protein**	**EC Number (b)**	**Score**	**Fold change**	**TRAT:CTRL_P**
Defense and virulence ROS					
PADG_01954	superoxide dismutase	1.15.1.1	2101.82	1.4	1
PADG_00324	catalase	1.11.1.6	5493.8	0.7	0
Membrane cell					
PADG_03199	ergosterol biosynthesis protein Erg 28		338.8	CTRL	
Fermentation					
PADG_11405	alcohol dehydrogenase 1	1.1.1.1	969.2	1.4	1

Figure S1. SDS-PAGE gel to assess the integrity of the proteins

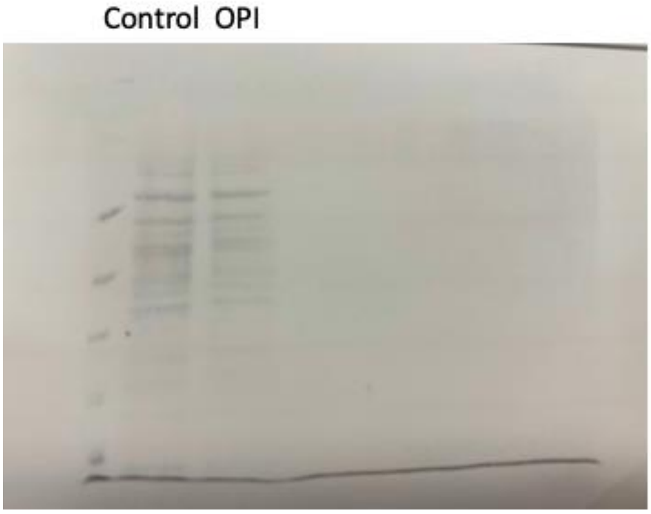

Supplement: Supplementary file 1 [file jof-09-00854-s001.zip › jof-2498666-supplementary.pdf]
